# Correction: Curcumin encapsulation in Pickering emulsions co-stabilized by starch nanoparticles and chitin nanofibers

**DOI:** 10.1039/d1ra90120f

**Published:** 2021-05-24

**Authors:** Yeong-Sheng Lee, Rodrigo Tarté, Nuria C. Acevedo

**Affiliations:** Department of Food Science and Human Nutrition, Iowa State University Ames IA 50011 USA nacevedo@iastate.edu +1-515-294-5962; Department of Animal Science, Iowa State University Ames IA 50011 USA

## Abstract

Correction for ‘Curcumin encapsulation in Pickering emulsions co-stabilized by starch nanoparticles and chitin nanofibers’ by Yeong-Sheng Lee *et al.*, *RSC Adv.*, 2021, **11**, 16275–16284, DOI: 10.1039/D1RA01622A.

The authors regret that the affiliations for the second (Rodrigo Tarté) and corresponding author (Nuria C. Acevedo) were incorrectly shown in the original article.

The correct affiliations are given here.

The Royal Society of Chemistry apologises for these errors and any consequent inconvenience to authors and readers.

## Supplementary Material

